# Intracranial Pressure Monitoring in Traumatic Brain Injury—A Tool of the Trade or One That Betrays Us?

**DOI:** 10.1001/jamanetworkopen.2023.34190

**Published:** 2023-09-05

**Authors:** Ruchira M. Jha

**Affiliations:** Neurology, Translational Science, Neurosurgery, Barrow Neurological Institute, Phoenix, Arizona.

The impact of intracranial hypertension on herniation and death in traumatic brain injury (TBI) has been recognized as far back as the Edwin-Smith Papyrus from ancient Egypt. The ability to measure intracranial pressure (ICP) directly within the cranium was developed in the 1950s. Subsequent research has demonstrated a causal relationship between elevated ICP and harm, forming the foundation for the now widespread role of ICP monitoring and management in TBI. The Brain Trauma Foundation (BTF) provides a Level IIB recommendation to use ICP data to decrease mortality.^1^ While the association between elevated ICP and TBI mortality has remained consistent over several studies and decades, methods of measurement, nuances of patient selection, and management of intracranial hypertension are more controversial—particularly given its unclear or inconsistent benefit for survivors. Standard methods of continuous ICP monitoring are invasive, and treatments are nonspecific with serious adverse effect profiles, raising questions about when, how, and in whom ICP should be measured after TBI—if at all.

Nattino et al^2^ evaluate the association of ICP monitoring with TBI outcomes using a propensity-score matched analysis in patients satisfying BTF criteria for monitoring in the CREACTIVE (Collaborative Research on Acute Traumatic Brain Injury in Intensive Care Medicine in Europe) Consortium database. The study compared 6-month Glasgow Outcome Scale-Extended scores between patients who underwent ICP monitoring within 48 hours of injury propensity-score matched to control individuals who were unmonitored or monitored after 48 hours. Of the 1448 patients included in the final analysis, 503 underwent ICP monitoring, confirming wide practice-pattern variations despite BTF guidelines. Propensity matching was rigorous, including 31 variables with a weighted postmatching analysis. A 1:1 matched design was not feasible given the propensity score distribution in the 2 groups, so a full-matching design was implemented where 1 monitored patient was matched with multiple control individuals or vice versa. There was no difference in mortality between monitored vs nonmonitored patients. However, ICP monitoring was associated with worse 6-month outcome, respiratory complications, infections, increased length of intensive care unit (ICU) stay, and duration of mechanical ventilation.

This is an important, well-designed, and thorough analysis in a large TBI cohort. However, propensity-matched studies are imperfect despite being a widely used tool to approximate causality. The authors acknowledge key limitations that may temper generalizability and preclude definitive conclusions regarding causative harm of ICP monitoring. Of 3154 eligible patients in the CREACTIVE database who met BTF criteria, fewer than half were analyzable. Moreover, the final cohort of 1448 patients represented 2 countries (Italy and Hungary) with the remaining patients excluded predominantly due to lack of high-quality within-country matching (n = 611) and 6-month outcomes (n = 389). Substantial between-country differences impact practice patterns and outcomes; here, 1174 patients (81% of the total cohort) were from Italy, 389 (33%) of whom had ICP monitoring. This contrasts with Hungary (approximately 19% of the total cohort), where the monitored-to-nonmonitored ratio was the inverse and approximately 60% of patients were monitored. Although addressed in their rigorous statistical analyses, prior to weighting, patients with ICP monitoring (n = 503) appeared sicker with proportionally more extracranial injuries (chest, spine), surgery or neurosurgery (before admission), cardiovascular failure, and petechiae on head computerized tomography scans vs the 945 control individuals. Whether the association between ICP monitoring and unfavorable outcome is mediated by therapeutic intensity level also remains unanswered.

Notwithstanding these unavoidable limitations, the findings by Nattino et al^2^ are provocative and propel discussion. Here, a measure that is supposed to be a means to an end may adversely impact the end itself. The results seemingly contradict those from SYNAPSE-ICU (Study on Intracranial Pressure in Intensive Care) where ICP monitoring was associated with more intensive therapies but lower 6-month mortality in a propensity-score analysis of 2395 patients (146 ICUs, 42 countries).^3^ Aside from methodological differences (fewer variables in propensity matching), importantly, SYNAPSE-ICU included patients with TBI (n = 1287), and an almost equal number (n = 1108) with nontraumatic intracranial and subarachnoid hemorrhage. This precludes direct comparisons. A more comparable prospective propensity-score matched analysis by Shibahashi et al^4^ included 31 660 patients with TBI from a nationwide database in Japan, 1907 of whom underwent ICP monitoring. Unlike Nattino et al,^2^ here 1:1 propensity-score matching using an extensive list of predetermined covariates was possible (likely given the large control sample size), with negligible imbalance. While both studies identified more frequent ICP-directed treatments and longer length of stay with ICP monitoring, Shibahashi et al^2^ reported a lower in-hospital mortality with monitoring (robust to sensitivity analyses)—particularly pronounced in patients with disorders of consciousness. A definitive conclusion is eluded since 6-month outcomes were not reported by Shibahashi et al, leaving the field open for debate. The short-term findings by Shibahashi et al may resonate with health care professionals who witness intracranial hypertension in TBI cause herniation and death. However, this does not negate important questions raised by Nattino et al regarding the potential impact of invasive monitoring and resultant aggressive therapies on longer-term outcomes.

Conceptually, similar questions have arisen in other realms of critical care monitoring. The pulmonary artery catheter, widely used in the 1970s to 1990s to obtain hemodynamic data (cardiac output, fluid responsiveness), fell out of favor after a landmark study^5^ demonstrated a 24% increased mortality risk with pulmonary artery catheter use. Key criteria contributing to the demise of pulmonary artery catheters include inaccurate data, catheter risks, harm from insufficient knowledge (interpretation and management), ubiquitous or minimally selective use, and overtreatment.^6^ These resonate strongly with current challenges of ICP monitoring in TBI and provide valuable lessons—especially regarding patient selection, data interpretation, and treatment. The question of whether to monitor is inextricably linked with monitoring methods and what we do with that information: who, when, and how do we treat?

Regarding who to monitor—the only randomized clinical trial comparing patients with TBI who were ICP monitored vs those who were not did not identify 6-month outcome differences between the groups.^7^ However, the mean and median percentages of readings demonstrating intracranial hypertension greater than 20 mm Hg were low: 20% and 7%, respectively. It is thus important to develop and validate biofluid and radiographic markers to better identify high-risk patient subgroups that may benefit from ICP monitoring and prevent unnecessary exposure in those unlikely to need it. Advancing the when and how of monitoring and/or treatment adds further complexity, but these questions are just as crucial. ICP values are less meaningful in isolation vs in the context of other parameters, like clinical or neurological examinations, temporal trajectories, burden of intracranial hypertension, waveform analyses, autoregulatory status, pressure reactivity indices, and optimal cerebral perfusion pressure. Practical and technological limitations have prevented several noninvasive methods from successfully replacing direct ICP measurement; however, ongoing research in this area is critical to reduce morbidity from monitoring. Since risks of ICP monitoring have, in part, been linked to complications of aggressive treatments, promising molecularly targeted treatments against cerebral edema (like glibenclamide) may reduce these adverse outcomes.

*Scientia potentia est*—knowing ICP in TBI can facilitate life-saving treatments. But is this superior to other clinical data? And how does monitoring and resultant treatment impact longer-term outcomes, particularly in survivors? The paradox of Schrodinger’s cat applies—intracranial hypertension may or may not be present but its extent in a patient who is comatose is known only when ICP is measured. If the possibility of greater harm suggested by Nattino et al^2^ is real and reproducible across populations, it bolsters the critical need to develop noninvasive methods of ICP monitoring, reevaluate our trigger for treatment, and advance better and more targeted therapies. Ultimately, ICP in isolation is a number. The real-world implications of this value require a clinical context. The true power to improve outcomes in patients with intracranial hypertension after TBI requires knowledge far beyond this number; monitoring ICP may be important, arguably necessary in some, but it is not sufficient.

## References

[1] Carney N, Totten AM, O’Reilly C, Guidelines for the management of severe traumatic brain injury, fourth edition. Neurosurgery. 2017;80(1):E3–E4. doi:10.1227/NEU.0000000000001432 27654000

[2] Nattino G, Gamberini L, Brissy O, ; CREACTIVE Consortium. Comparative effectiveness of intracranial pressure monitoring on 6-month outcomes of critically ill patients with traumatic brain injury. JAMA Netw Open. 2023;6(9):e2334214. doi:10.1001/jamanetworkopen.2023.34214 37755832 PMC10534270

[3] Robba C, Graziano F, Rebora P, ; SYNAPSE-ICU Investigators. Intracranial pressure monitoring in patients with acute brain injury in the intensive care unit (SYNAPSE-ICU): an international, prospective observational cohort study. Lancet Neurol. 2021;20(7):548–558. doi:10.1016/S1474-4422(21)00138-1 34146513

[4] Shibahashi K, Ohbe H, Matsui H, Yasunaga H. Real-world benefit of intracranial pressure monitoring in the management of severe traumatic brain injury: a propensity score matching analysis using a nationwide inpatient database. J Neurosurg. 2023;(June):1–9. doi:10.3171/2023.4.JNS23146 37310047

[5] Connors AF Jr, Speroff T, Dawson NV, ; SUPPORT Investigators. The effectiveness of right heart catheterization in the initial care of critically ill patients. JAMA. 1996;276(11):889–897. doi:10.1001/jama.1996.03540110043030 8782638

[6] Marik PE. Obituary: pulmonary artery catheter 1970 to 2013. Ann Intensive Care. 2013;3(1):38. doi:10.1186/2110-5820-3-38 24286266 PMC4175482

[7] Chesnut RM, Temkin N, Carney N, ; Global Neurotrauma Research Group. A trial of intracranial-pressure monitoring in traumatic brain injury. N Engl J Med. 2012;367(26):2471–2481. doi:10.1056/NEJMoa1207363 23234472 PMC3565432

